# Composite body movements modulate numerical cognition: evidence from the motion-numerical compatibility effect

**DOI:** 10.3389/fpsyg.2015.01692

**Published:** 2015-11-05

**Authors:** Xiaorong Cheng, Hui Ge, Deljfina Andoni, Xianfeng Ding, Zhao Fan

**Affiliations:** ^1^Key Laboratory of Adolescent Cyberpsychology and Behavior (CCNU), Ministry of EducationWuhan, China; ^2^School of Psychology, Central China Normal University (CCNU)Wuhan, China; ^3^Department of Public Education, Tibet Vocational Technical CollegeLhasa, China

**Keywords:** numerical cognition, composite body movements, lateral head/arm turns, random number generation, situated cognition

## Abstract

A recent hierarchical model of numerical processing, initiated by Fischer and Brugger (2011) and Fischer (2012), suggested that situated factors, such as different body postures and body movements, can influence the magnitude representation and bias numerical processing. Indeed, Loetscher et al. (2008) found that participants’ behavior in a random number generation task was biased by head rotations. More small numbers were reported after leftward than rightward head turns, i.e., a motion-numerical compatibility effect. Here, by carrying out two experiments, we explored whether similar motion-numerical compatibility effects exist for movements of other important body components, e.g., arms, and for composite body movements as well, which are basis for complex human activities in many ecologically meaningful situations. In Experiment 1, a motion-numerical compatibility effect was observed for lateral rotations of two body components, i.e., the head and arms. Relatively large numbers were reported after making rightward compared to leftward movements for both lateral head and arm turns. The motion-numerical compatibility effect was observed again in Experiment 2 when participants were asked to perform composite body movements of congruent movement directions, e.g., simultaneous head left turns and arm left turns. However, it disappeared when the movement directions were incongruent, e.g., simultaneous head left turns and arm right turns. Taken together, our results extended Loetscher et al.’s (2008) finding by demonstrating that their effect is effector-general and exists for arm movements. Moreover, our study reveals for the first time that the impact of spatial information on numerical processing induced by each of the two sensorimotor-based situated factors, e.g., a lateral head turn and a lateral arm turn, can cancel each other out.

## Introduction

Recent studies (for a recent review see Fischer and Shaki, 2014) suggested that our numerical processing is more than constructing and operating abstract concepts and internal representations that are independent from constraints of the physical world, the body and the situated context. Instead, it is deeply rooted in and consistently shaped by specific motor activities and sensory-bodily experiences and can be dynamically altered by contextual requirements of a task (Fischer and Brugger, 2011; Fischer, 2012). For example, Loetscher et al. (2008) asked participants to report random numbers in a range of 1–30 with responses paced by a metronome (0.5 Hz). They found that participants’ performance of verbally reporting numbers ‘at random’ in a random number generation (RNG) task was systematically influenced by lateral head movements (Loetscher et al., 2008). More specifically, relatively large numbers were reported after head turns to the right, whereas relatively small numbers were reported after lateral head turns to the left, i.e., a motion-numerical compatibility effect. The robustness of this effect was confirmed by a later study (Winter and Matlock, 2013) along both horizontal and vertical dimensions.

The motion-numerical compatibility effect induced by head rotations was generally explained as that head movements provide spatial cues and shift attention along a hypothetical “mental number line (MNL),” which in turn bias the accessibility of numerical representation during a RNG task (Loetscher et al., 2008). This explanation is consistent with the MNL hypothesis that human represents the magnitude of numbers on a spatially oriented mental line, where small/large numbers are located on the left/right side of the body and accessed by mechanisms based on spatial attention (for a review see Hubbard et al., 2005). Dehaene et al. (1993) proposed this hypothesis initially to explain the effect of spatial-numerical association of response codes (SNARCs), which reflects a robust pattern of reacting faster to large numbers with right-side responses and to small numbers with left-side responses. Studies on the SNARC effect and many other studies on numerical cognition in recent decades (for a recent review see Fischer and Shaki, 2014) contributed together to the notion that our numerical processing is closely linked with activation of spatial information, i.e., spatial-numerical associations (SNAs).

A recent theoretic development, initiated by Fischer and Brugger (2011) and Fischer (2012), put the origins of the SNAs and the effects of sensorimotor experience on numerical processing in a frame work of grounded, embodied, and situated numerical cognition (see General Discussion for more details of this model). According to this hierarchical model of numerical processing, situated factors, such as different body postures in a magnitude estimation task (Eerland et al., 2011) and head movements in a RNG task (Loetscher et al., 2008), can dynamically influence the magnitude/number representation and thus produce biases during numerical processing. An interesting prediction following this model is that any body movement, as long as it can activate spatial representations, will potentially influence numerical processing and produce biases in a RNG task. In other words, the effect revealed by Loetscher et al. (2008) in a RNG task should be effector-general. The term ‘effector-general’ here means that the numerical biases in a RNG task is not specific for movements of the head but also can be found for movements of other important body components, for example arms.

However, the notion that the motion-numerical compatibility effect is effector-general was supported from only three findings so far. First, it was found that the directions of eye movements were systematically correlated with the changes of number sizes during random number production (Loetscher et al., 2010). Second, a later study (Hartmann et al., 2012) demonstrated that the reported numerical magnitudes in a RNG task were systematically influenced by the directions of passive whole-body motions. This finding suggested that the motion-numerical compatibility effect does not rely on moving a specific body part such as the head or the eyes in isolation. Instead, the bottom–up vestibular activation without movements of a single body part can induce a similar motion-numerical compatibility effect. Third, in a recent study, Shaki and Fischer (2014) demonstrated that the directions of left/right turns during random walks are closely associated with the numerical biases in a RNG task. To the best of our knowledge, there was no study so far to systematically explore whether similar motion-numerical compatibility effects can be observed for movements of an important body component, i.e., arms.

There are several reasons driving our interest on movements of arms. First, many studies have revealed a close relationship between arm/hand-based sensorimotor activities and numerical processing, including grasping (Andres et al., 2004; Badets et al., 2007, 2010, 2012; Badets and Pesenti, 2010), pointing movements (Fischer, 2003), observations of visually mimicked human pointing movements (Badets et al., 2015), and finger counting (Fischer and Brugger, 2011). However, the association between numerical magnitudes and grasping/pointing/finger counting was largely established based on perceptual and/or static cues (e.g., gestures of hands and apertures of palms), local movements in a small scale (e.g., finger bending and palm closing), or observations of arm or hand movements. It is not yet established whether physical movements of arms and hands in a large magnitude, such as lateral turns of arms and hands relative to the body trunk, also bias internal numerical representation in a RNG task. Second, there seems to be only one study (Wiemers et al., 2014) that demonstrated how continuous arm movements modulate numerical processing. This study indicated that arm movements and number magnitude processing are intrinsically linked in a mental arithmetic task. This is a so called motion-arithmetic compatibility effect, i.e., the arithmetical calculations became easier when the type of calculation and the direction of the arm movements were congruent (right/upward motion-additions; left/downward motion-subtractions) than when they were incongruent (right/upward motion- subtractions; left/downward motion- additions). However, it is worth noting that Wiemers et al. (2014) emphasized how arm movements modulate spatial processing on *arithmetical calculations*, e.g., addition and subtraction, rather than on representation of single numbers, e.g., RNG. Thus, the evidence for the existence of a motion-numerical compatibility effect on arm movements is rather indirect, and a strong test of the coupling of space and numerical representations, such as an effect of arm movements on the performance in RNG tasks, is still missing. This leaves us space for exploration (see Discussion of Experiment 1 for more on this issue). Third, we argue that lateral head turns activate the proprioceptive system as well as the vestibular system, while lateral arm turns activate the proprioceptive system dominantly. Thus, studies on comparing lateral arm movements and lateral head movements may shed some light on the specific role of the proprioceptive system on numerical cognition (see General Discussion for more on this issue).

**FIGURE 1:**
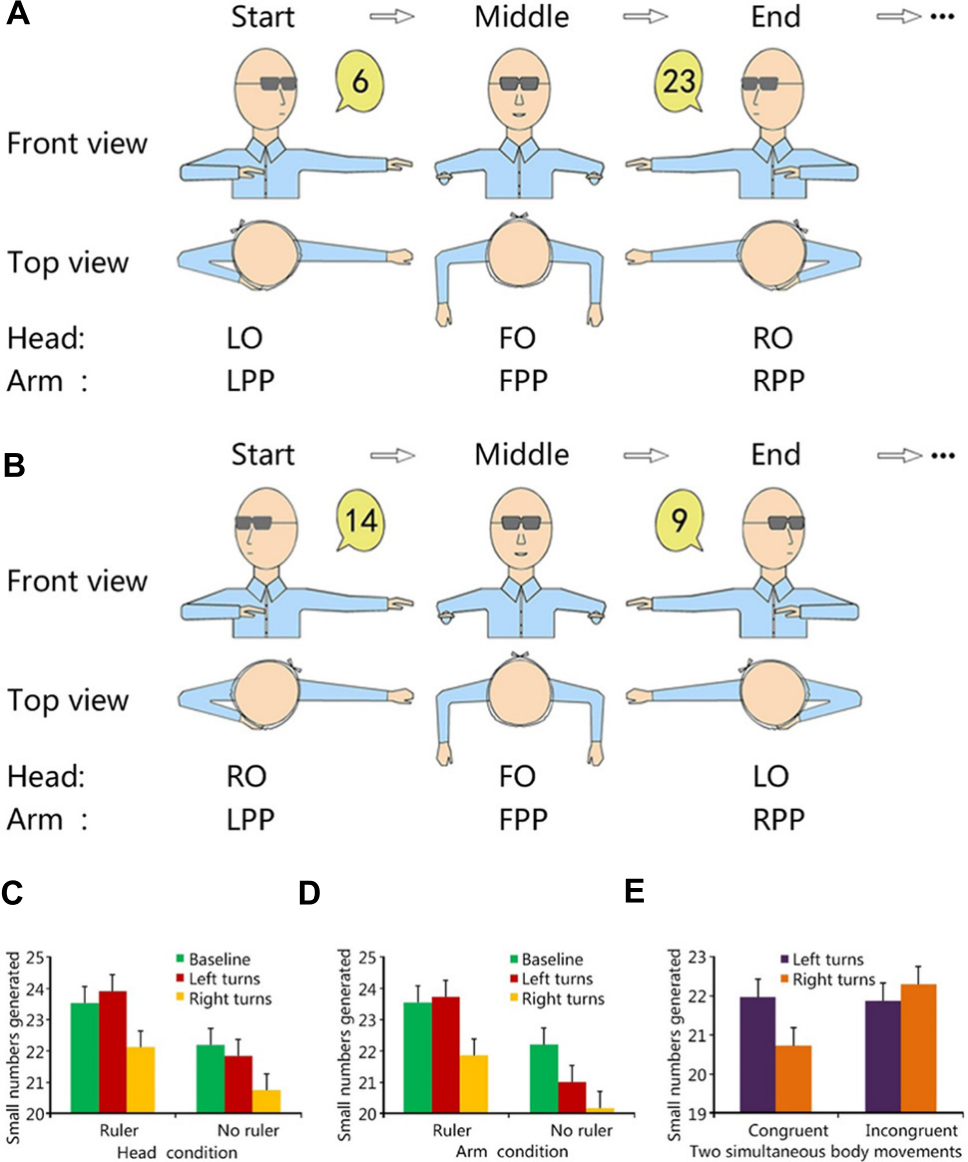
**Experimental approach and results of Experiments 1 and 2.** **(A)** Congruent condition in Experiment 2. **(B)** Incongruent condition in Experiment 2. Note, Experiment 1 used a similar approach as in Experiment 2 except that the arm turns and the head turns were in different conditions. The start, middle and end postures of one lateral head/arm turn were labeled. Two perspectives, i.e., Frontal view and Top view, were used. **(C)** Results of the head turn condition in Experiment 1. **(D)** Results of the arm turn condition in Experiment 1. **(E)** Results of Experiment 2 (The ‘Left turns’ and ‘Right turns’ refer to the lateral turns with their directions defined by the head turns). Error bars are within-subjects SEs (Cousineau, 2005). LO, left orientation; FO, front orientation; RO, right orientation; LPP, left pointing position; FPP, front pointing position; and RPP, right pointing position.

We designed an ‘arm turns’ condition (more details see Methods of Experiment 1 and arm movements in **Figures 1A,B**) in Experiment 1 of the present study and used another condition, i.e., ‘head turns,’ which was a detailed replication of Loetscher et al.’s (2008) paradigm, including the number range and the response pace. The aim of Experiment 1 is to investigate whether the effect revealed by Loetscher et al. (2008) is effector-general and exists for movements of other important body components (i.e., arms, which are heavily involved in numerical cognition in the current literature) as well. Our paradigm should help to further describe the nature of the spatial bias in RNGs.

Experiment 2 is a natural extension of Experiment 1. Each of the two conditions in Experiment 1 involved a single sensorimotor-based situated factor, i.e., either head turns or arm turns. In daily life we frequently rotate our head or turn arms in response to specific situation of the environment and/or particular context of a task. For example, a recent study demonstrated that arm/hand gestures were frequently used in situations outside constrained laboratory settings, such as our daily communication, to express magnitude information (Winter et al., 2014). Thus, a natural follow-up question from Experiment 1 is to ask whether the impact of spatial information on numerical processing induced by each of the two sensorimotor-based situated factors could interact with each other. To our knowledge, so far there is no literature to tackle this question directly, which provides us a strong rationale to perform further exploration. Besides, this question also has practical implications. In many situations of daily living, such as dancing or traffic policing, movements of one body part, such as lateral arm turns, are frequently accompanied by movements of another body part, such as lateral head turns. In some occasions, the directions of lateral movements of the head and the arms are congruent (**Figure 1A**), while in other occasions they are incongruent (**Figure 1B**). For example, all natural orienting responses are usually congruent head and body movements, whereas some compensatory postural reflexes involve incongruent body movements, e.g., leaning back while reaching forward.^1^ An interesting question would be whether one body movement has priority and can mask the effect of another body movement. For example, in situations when the directions (left and right) of lateral turns of two body components (head, arms) are opposite, which body component will decide the direction of a sensorimotor-related numerical bias (a larger or a smaller number production in a RNG task)?

Thus, Experiment 2 in the present study tries to extend the role of body movements into more ecologically meaningful situations when multiple body movements and their related sensorimotor inputs are present. In a complex scenario, such as random walk (Shaki and Fischer, 2014), multiple body movements of the head, arms, hands, legs and trunk and their related sensorimotor processing, including body posture control, coordination of proprioceptive, and vestibular inputs etc., are activated simultaneously. In order to tackle our specific question, a simplified scenario may be suitable. In Experiment 2, we designed a new paradigm (see **Figures 1A,B**) for such a simplified scenario, i.e., composite body movements consisting of two sensorimotor-based situated factors- a lateral head turn and a lateral arm turn. The aim of Experiment 2 is to investigate whether and how composite body movements that contain two sensorimotor-based situated factors can modulate numerical cognition, and whether the effects of the two situated factors can interact with each other.

## Experiment 1

In Experiment 1 we designed an ‘arm turns’ condition. For conciseness of narration, we used the term ‘arm turns’ to name the experimental condition which involves lateral movements of both forearms and hands since hands are natural extensions of two forearms. The other condition, i.e., ‘head turns’ was a replication of Loetscher et al.’s (2008) paradigm. We expected an effect of the compatibility between the directions of the arm movements and the reported number magnitudes in the RNG task. That is, based on studies on the similar effect on head movements (Loetscher et al., 2008) a motion-numerical compatibility effect should be reflected by generating more small numbers for a leftward rather than rightward arm movement.

### Method

#### Participants

Sixty-four healthy, right-handed paid participants (21 males and 43 females) from Central China Normal University participated in the experiment. All participants signed a consent form according to the requirements of Institutional Review Board of CCNU. They were 21.7 years-old on average (range: 18–28). All participants were naive to the purpose of the experiment. It is worth to note that all the participants in the present study were mainland-Chinese. A recent study (Yang et al., 2014) with 314 mainland-Chinese children (from kindergartens to 6th grades) and adults demonstrated that all age groups of these participants showed a significant (marginally for 1st graders) SNARC effect with the same direction (small numbers associated to the left and large numbers associated to the right) as Western participants (Dehaene et al., 1993). It implied that mainland-Chinese already develop spatial representations of numbers with the same directions as Westerners as early as in the preschool. Thus, we do not expect the effect of lateral head turns of the present study deviate from Loetscher et al.’s (2008) study (with Westerner participants) due to cultural differences in the representation of numbers.

#### Procedure

Participants were required to report numbers between 1 and 30 as randomly as possible with their eyes covered by a mask in three blocks, i.e., head turns, arm turns and a baseline condition. All participants took part in both head turn and arm turn blocks (order counterbalanced across participants) before the baseline condition without any body movement. In the baseline condition, 40 responses were generated while keeping the head straight and the arms/hands stationary in natural positions, vertical to the ground. Responses were paced by a metronome (0.5 Hz).

In the block of head turns, participants were asked to orally report random numbers while performing periodic lateral head turns in the yaw plane between two maximum rotation angles of turning head to the left and to the right as far as possible (see **Figure 1A** head turn for a graphical description). For conciseness of narration, we used the terms ‘Left Orientation (LO)’ and ‘Right Orientation (RO)’ to denote these two maximum rotation angles that were constrained by biomechanical properties of the human neck (Snyder et al., 1975). We also used the term ‘Front Orientation (FO)’ to denote the head position when the head was straight. Half the participants started from the left side (LO), and the other half started from the right side (RO). The lateral head turns were symmetric. Thus, for the participants who started from the left side, the trajectory of the head covered a periodic sequence of LO-FO-RO-FO-LO-FO-RO-FO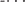 and so on. For the participants who started from the right side, the trajectory of the head covered a periodic sequence of RO-FO-LO-FO- RO-FO-LO-FO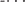 and so on. The participants were asked to orally report numbers randomly when their heads were in positions of the LO or RO only with responses paced by a metronome (0.5 Hz), i.e., the same procedure as that in Loetscher et al. (2008). Eighty responses- 40 to the left (LO) and 40 to the right (RO) -were collected.

In the block of arm turns, both upper arms of the participants were kept stationary (to minimize motion of the trunk), parallel with the ground. All the fingers were straightened and laterally closed during arm turns. The participants were asked to orally report random numbers while performing periodic lateral turns of both forearms and hands in a body-transverse plane continuously across three key arm postures with their eyes covered by a mask (to cut off visual feedback). We denoted the first arm posture as ‘Left Pointing Position (LPP)’ where the left arm and hand (including forearm, upper arm, and hand) and the right forearm and hand were outstretched by pointing to the left side, parallel to the ground floor, and perpendicular to the body-midsagittal plane. The positions of the two hands differed in that the left hand was stretched further away from the midline of the body while the right hand was close to it though both hands pointed to the same direction of the external space (see **Figure 1A** arm turn for a graphical description). In this arrangement, both the rotating direction and the pointing orientation were consistent with the side where the hand stretched further away. The second arm posture was denoted as ‘Right Pointing Position (RPP)’ where the right arm and hand (including forearm, upper arm, and hand) and the left forearm and hand were outstretched by pointing to the right side, parallel to the ground floor, and perpendicular to the body-midsagittal plane. Similarly, the two hands were positioned asymmetrically, i.e., the right hand was stretched further away from the midline of the body and the left hand was close to the midline though both hands pointed to the right (see **Figure 1A** arm turn for a graphical description). The third arm posture was denoted as ‘Front Pointing Position (FPP)’ where both the left and the right forearms were outstretched by pointing to the front side, parallel to the ground floor, and perpendicular to the body-mid-coronal plane (see **Figure 1A** arm turn for a graphical description).

In the block of arm turns, half the participants started from the left side (LPP), and the other half started from the right side (RPP). The lateral arm turns were symmetric. Thus, for the participants who started from the left side, the trajectory of the arms covered a periodic sequence of LPP-FPP-RPP-FPP-LPP-FPP-RPP-FPP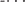 and so on. For the participants who started from the right side, the trajectory of the arms covered a periodic sequence of RPP-FPP-LPP-FPP- RPP-FPP-LPP-FPP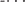 and so on. The participants were asked to orally report random numbers only when their arms were in the LPP or RPP but no other positions with a response frequency of 0.5 Hz paced by a metronome. Eighty responses- 40 to the left (LPP) and 40 to the right (RPP) -were collected. Both hands and forearms of the participants performed similar semi-circular rotations pivoted at each elbow joint. The synchronized rotations should have canceled any potential contralateral hemispheric activation (since each arm is controlled by the contralateral hemisphere) if it occurred. Following Loetscher et al.’s (2008) study, participants’ visual feedback was cut off while performing the arm turns as well as the head turns.

We also included a manipulation of ‘ruler imagery instruction’ (the same manipulation as that in Loetscher et al., 2008) in this experiment. Thirty two participants were told that the imagination of a ruler with 30 units might facilitate performance (ruler group). No such information was given to the other 32 participants (no ruler group). The purpose of this manipulation was to investigate whether visual imagery instructions can influence information accessibility along the MNL during arm turns, i.e., similar to that found in a paradigm of head turns (Loetscher et al., 2008).

### Results

Numbers of ‘small’ numbers (< = 15) generated under the baseline, the head turn and the arm turn conditions were calculated, respectively. Participants produced more small numbers than expected by chance (20 out of 40 responses) in conditions of the baseline [*t*(63) = 5.873, *p* < 0.001, *M* = 22.86; *SD* = 3.89], the head left turns [*t*(63) = 6.84, *p* < 0.001, *M* = 22.88; *SD* = 3.36], the head right turns [*t*(63) = 3.08, *p* < .004, *M* = 21.44; *SD* = 3.73] and the arm left turns [*t*(63) = 4.83, *p* < 0.001, *M* = 22.36; *SD* = 3.91], supporting an overall small number bias (SNB) during RNG (Loetscher and Brugger, 2007). Only in the condition of the arm right turns, small number generations were not significantly different from the chance level [*t*(63) = 1.96, *p* = 0.054, *M* = 21; *SD* = 4.07].

A repeated-measure ANOVA with two within-subjects variables, body movement (baseline-no body movement, left turns, or right turns) and body part (head or arm), and one between-subject variable, instruction group (ruler or no ruler), revealed a significant main effect of body movement [*F*(2,124) = 7.16, *p* = 0.001, η^2^ = 0.104] and a significant main effect of instruction group as well [*F*(1,62) = 6.27, *p* = 0.015, η^2^ = 0.092] (**Figures 1C,D**). No other main effect or any interactions was significant (*p*s > 0.05). In terms of body movement, participants generated significantly more small numbers after left body (head or arm) turns than after right body turns (average difference = 1.398, *SE* = 0.456, *p* = 0.01, after Bonferroni correction), mirroring the effect of lateral head turns revealed by Loetscher et al.’s (2008) research. Participants generated significantly more small numbers in the baseline condition than after right body turns (average difference = 1.641, *SE* = 0.46, *p* = 0.002, after Bonferroni correction). There was no significant difference between conditions of the baseline and left body turns (average difference = 0.242, *SE* = 0.49, *p* ≈ 1.0, after Bonferroni correction). For the effect of different instruction groups, the ‘ruler instruction’ group produced more small numbers than the ‘no-ruler instruction’ group (average difference = 1.755, *SE* = 0.70, *p* = 0.015, after Bonferroni correction). Since neither the main effect of body part (head or arm, *p* = 0.11) nor all interactions concerning body part were significant, the arms had a similar role as the head in the body-movement-induced numerical bias.

### Discussion

Experiment 1 demonstrated a typical pattern of motion-numerical compatibility effect induced by body movements in that a left-lateral turn of body parts, either the head (**Figure 1C**) or the arms (**Figure 1D**), facilitated more small number generations relative to a right-lateral turn. This motion-numerical compatibility effect indicated that body movements of the head or the arms provide spatial cues and shift attention along the horizontal axis with small numbers on the left and large number on the right. Thus, our findings extended Loetscher et al.’s (2008) finding by demonstrating that the effect revealed by Loetscher and colleagues is effector-general. The numerical biases induced by body movements were widely present not only in lateral head turns, but also in lateral arm turns. Our instructions emphasized that participants should keep their head straight and still in the arm turn condition. We therefore argue that the head did not play a crucial role in the arm turn condition. Nevertheless we acknowledge that in principle it cannot be excluded that minor head movements (and resulting vestibular stimulation) might have contributed partially to the observed effect of arm movements, since we did not provide physical constraints and/or movement monitoring to the participants’ head. This issue is upon further investigation with a more strictly controlled procedure.

Mirroring Loetscher et al.’s (2008) results in , the visual imagery instruction is a potent means of exaggerating small-number preferences. Participants who imagined a ruler showed a more pronounced preference for small numbers than participants in the no-ruler group who conceived of the numbers in a more abstract way, suggesting a spatial feature of the number representation. Moreover, this effect was observed for both body parts (head or arms), indicating that the effect of visual imagery instructions on information accessibility along the presumed MNL is effector-general.

Our results in Experiment 1 were consistent with a recent study indicating that motor actions, specifically arm movements, and number magnitude processing are intrinsically linked (Wiemers et al., 2014). In Wiemers et al.’s (2014) study , participants were required to solve addition and subtraction problems and reported results verbally while moving their outstretched right arm continuously left-, right-, up-, or downward. A motion-arithmetic compatibility effect was found and this study provided the first evidence for an impact of spatial processing on mental arithmetic. It is worth to note that Wiemers et al. (2014) emphasized on how sensorimotor activity modulates spatial processing on arithmetical calculations rather than on the represntation of single numbers. Here, by using the RNG paradigm, our study asked a different question, i.e., whether arm movement-induced activation of spatial codes can modulate the representation of single numbers rather than mental arithmetic. This question is worth of examination for two reasons. First, two recent studies (Pinhas et al., 2014; Hartmann et al., 2015) demonstrated that a semantic component, i.e., the semantic association between operation signs (e.g., operators: ‘+’ vs. ‘-’) and space, rather than the merely activated magnitude of the solution of an arithmetic problem plays a dominant role on spatial biases during mental arithmetic. Thus, it is unclear to what extent the reported results in a motion-arithmetic compatibility effect reflect spatial biases induced by the actual numerical representations (e.g., the activated magnitudes) rather than by the semantic spatial association of the operation sign (i.e., the semantic component). Therefore, whether arm movements can modulate the representation of single numbers in a RNG task without such a semantic component (Pinhas et al., 2014; Hartmann et al., 2015), is still subject to empirical examination. Second, our study tried to explore the SNA at the level of the single number representation and our finding may provide evidence for theories of more complex numerical tasks, such as mental arithmetic, in which the operation sign spatial association (OSSA; Pinhas et al., 2014) and magnitude activations at multiple levels are likely to be involved simultaneously (McCrink et al., 2007).

Experiment 1 provided evidence for an impact of spatial processing on the representation of single numbers by showing a systematic effect of movement direction of head turns as well as arm turns on RNG performance. Next, we conducted a second experiment in order to explore whether composite body movements can modulate numerical cognition. In particular, we were interested in the fate of the motion-numerical compatibility effect when participants were asked to perform composite body movements in congruent directions as well as in incongruent directions.

## Experiment 2

In Experiment 2, we tried to explore the role of different body components (head vs. arms) and their related sensorimotor activities in more ecologically meaningful situations, i.e., composite body movements. In this experiment, participants were requested to perform in two conditions: a congruent and an incongruent condition of simultaneous head and arm movements. In the congruent condition (**Figure 1A**), the head and arms turned simultaneously in the same direction (e.g., a head left turn plus arm left turn), while in the incongruent condition (**Figure 1B**) the head and arms turned simultaneously in the opposite directions (e.g., a head left turn plus arm right turn). We would expect to observe a similar motion-numerical compatibility effect as in Experiment 1 for the condition of ‘congruent’ body movements. Regarding possible outcomes for the condition of ‘incongruent’ body movements, there were three possibilities. First, it could be that the effect of lateral head turns dominates and can mask the effect of lateral arm turns. If this occurred, the direction of RNG (a larger or a smaller number production) is decided by the direction (left or right) of head movements. A second potential result was that the effect of lateral arm turns dominates and can mask the effect of lateral head turns. If that was true, we would expect to observe that the direction of RNG is decided by the direction of arm movements instead. The last potential result in the incongruent condition was that the numerical biases from the lateral head turns and that from the lateral arm turns cancel each other out.

### Methods

#### Participants

Thirty healthy, right-handed paid participants (9 males and 21 females) from Central China Normal University participated in the experiment. All participants signed a consent form according to the requirements of Institutional Review Board of CCNU. They were 23.3 years-old on average (range: 18–28). All participants were naive to the purpose of the experiment.

#### Procedure

Participants were required to perform two blocks in sequence with their eyes covered by a mask. In one block, participants were requested to move their head and arms in the opposite directions spontaneously, i.e., an incongruent condition. Half participants started with a head left plus arm right turn, the other half started with a head right plus arm left turn. The lateral turns of the head and of the arms were always in antiphase. Thus, for the participants who started with a head left plus arm right turn, the trajectory of the head (arms) covered a periodic sequence of LO (RPP)-FO (FPP)-RO (LPP)-FO (FPP)-LO (RPP)-FO (FPP)-RO (LPP)-FO (FPP)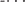 and so on. For the participants who started from a head right plus arm left turn, the trajectory of the head (arms) covered a periodic sequence of RO (LPP)-FO (FPP)-LO (RPP)-FO (FPP)-RO (LPP)-FO (FPP)-LO (RPP)-FO (FPP)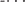 and so on. The participants were asked to orally report random numbers when their heads (arms) were in positions of the LO (RPP) or RO (LPP) only with a response frequency of 0.5 Hz paced by a metronome.

The other block was called as the congruent condition in which participants moved their head and arms in the same direction spontaneously. Half participants started with a head left plus arm left turn, and the other half started with a head right plus arm right turn. The lateral turns of the head and of the arms were always in phase. Thus, for the participants who started with a head left plus arm left turn, the trajectory of the head (arms) covered a periodic sequence of LO (LPP)-FO (FPP)-RO (RPP)-FO (FPP)-LO (LPP)-FO (FPP)-RO (RPP)-FO (FPP)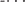 and so on. For the participants who started from a head right plus arm right turn, the trajectory of the head (arms) covered a periodic sequence of RO (RPP)-FO (FPP)-LO (LPP)-FO (FPP)-RO (RPP)-FO (FPP)-LO (LPP)-FO (FPP)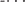 and so on. The participants were asked to orally report random numbers when their heads (arms) were in positions of the LO (LPP) or RO (RPP) only with a response frequency of 0.5 Hz paced by a metronome.

The order of the two blocks (congruent, incongruent) was counterbalanced across participants. In each block, 80 responses of RNGs- 40 to head left turns and 40 to head right turns -were collected. No participant encountered problems when performing the congruent and the incongruent blocks or reported task demand difference across blocks when asked at the end of the experiment.

### Results

Numbers of ‘small’ numbers (< = 15) generated under conditions^2^ of the congruent head left, the congruent head right, the incongruent head left and the incongruent head right were calculated, respectively. Participants produced more small numbers than expected by chance (20 out of 40 responses) in conditions of the congruent head left [*t*(29) = 3.05, *p* < 0.006, *M* = 21.97; *SD* = 3.54], the incongruent head left [*t*(29) = 2.96, *p* < 0.007, *M* = 21.87; *SD* = 3.45] and the incongruent head right [*t*(29) = 3.6, *p* < 0.002, *M* = 22.3; *SD* = 3.5], supporting an overall SNB during RNG (Loetscher and Brugger, 2007). However, in condition of the congruent head right, small number generation was not significantly different from chance level [*t*(29) = 1.34, *p* = 0.192, *M* = 20.73; *SD* = 3.0].

A repeated-measure ANOVA with two within-subject variables, the direction of head turns (left, right) and the directional congruence between head turns and arm turns (congruent, incongruent) revealed a significant interaction of the two factors [*F*(1,29) = 4.885, *p* < 0.05, η^2^ = 0.144] (**Figure 1E**). When the directions of turns were the same, i.e., in the congruent condition, left turns induced more small numbers than right turns (average difference = 1.233, *SE* = 0.590, *p* < 0.05, after Bonferroni correction], while there was no such difference (*p* = 0.368) when the lateral turn directions were opposite, i.e., the incongruent condition. The main effects of both the direction of body turns [with reference to the head turn direction; *F*(1,29) = 1.116, *p* = 0.299] and the directional congruence between head turns and arm turns [*F*(1,29) = 1.126, *p* = 0.297] failed to reach the significant level.

### Discussion

The motion-numerical compatibility effect was observed in Experiment 2 (**Figure 1E**) when participants were asked to perform composite body movements and the directions of head and arm turns were congruent, e.g., simultaneous head left turns and arm left turns. However, it disappeared when the directions of head and arm turns were incongruent, e.g., simultaneous head left turns and arm right turns. Taken together, the results of Experiment 2 suggest that composite body movements, such as simultaneous lateral turns of both the head and arms, modulated the brain’s internal numerical processing. Head turns did not play a dominant role in situations involving incongruent directions of body movements. Instead, the numerical biases from the lateral head turns and that from the lateral arm turns canceled each other in the incongruent condition. In our view, this is strong evidence that the impact of spatial information on numerical processing induced by each of the two sensorimotor-based situated factors, e.g., a lateral head turn and a lateral arm turn, can cancel each other out.

Experiment 2 tried to explore the motion-numerical compatibility effect in more ecologically meaningful situations, i.e., composite body movements. A similar motion-numerical compatibility effect was also unfolded in situations outside of constrained laboratory settings, such as our daily communication when we try to use arm/hand gestures to express magnitude information. More specifically, by analyzing 552 video footages from television newscasts, a recent study (Winter et al., 2014) demonstrated that human can use naturally occurring gestures to describe numbers and quantities. It was revealed that a speaker’s two forearms/hands during natural gesture production mimic known spatial mappings between space and quantity. These spatial mappings include “more is to the right” (e.g., a speaker sweeps his forearm/hand from left to right during talking about a number increase), “more is up” (e.g., a speaker’s left forearm/hand starts at the stomach level and raises to the shoulder level during mentioning a larger number) and “more is big” (e.g., a speaker makes an outward-oriented gesture during mentioning a bigger number). This study implied robustness of the motion-numerical compatibility effect in the situations of spontaneous communication without strict experimental control.

## General Discussion

Our study contributed to the idea that numerical cognition is profoundly influenced by sensorimotor activities by demonstrating that basic units of sophisticated body movements, e.g., lateral head/arm turns and their combinations, provide sensorimotor-based modulation on human numerical cognition. By carrying out two experiments we replicated and extended Loetscher et al. (2008) finding that sensorimotor activities systematically bias the brain’s internal random number generator. In Experiment 1, a motion-numerical compatibility effect was observed for lateral rotations of two body components, i.e., the head and the arms. Relatively large numbers were reported by participants after making rightward compared to leftward movements for both lateral head turns and lateral arm turns. This finding suggested that the effect revealed by Loetscher et al. (2008) is effector-general and can be observed for other body movements, for example, lateral arm turns. A typical motion-numerical compatibility effect was observed again in Experiment 2 when participants were asked to perform composite body movements and the directions of head and arm turns were congruent, e.g., simultaneous head left turns and arm left turns. However, it disappeared for other composite body movements when the directions of head and arm turns were incongruent t, e.g., simultaneous head left turns and arm right turns. This effect is likely due to the fact that the composite body movements of incongruent directions tended to shift attention into opposite directions along a horizontally oriented MNL. The outcome was that the execution of the attentional shifts along opposite directions cancel each other out eventually. These findings added to the rapidly accumulating evidence supporting the idea that our numerical cognition is deeply rooted in specific motor activities and sensor-body experiences.

The present study provided an ideal testing ground for research on grounded, situated and embodied numerical cognition and provided new evidence for the hierarchical model of numerical processing, initiated by Fischer and Brugger (2011) and Fischer (2012). First, our results supported the notion that situated factors, either task-required or context-constrained, can modulate numerical cognition. More importantly, our study demonstrated for the first time that the impact of spatial information on numerical processing induced by two sensorimotor-based situated factors, e.g., a lateral head turn and a lateral arm turn, can cancel each other out. Below, we tried to extend and discuss the implications of our findings in the context of the current literature.

### The Situated Nature of Spatial-numerical Associations

Recently, Fischer and Brugger (2011) and Fischer (2012) proposed a hierarchical model of grounded, embodied, and situated numerical cognition. According to this theory, grounded cognition serves as the most fundamental aspect of magnitude/number representations. The concept of grounded cognition refers to the idea that universal laws of the physical world, such as small magnitude/numbers are associated with lower space whereas large magnitude/numbers are associated with upper space (Lakoff and Johnson, 1980), are reflected in our magnitude/number representation. Embodied cognition is built on the basis of grounded cognition and emphasizes sensorimotor (embodied) knowledge representations that are acquired during repeated, culturally dependent learning, such as reading and writing directions (Zebian, 2005; Shaki et al., 2009) and finger counting habits (Fischer and Brugger, 2011). Finally, situated cognition describes the flexibility of magnitude/number representations and refers to the idea that task-specific constraints, such as different body postures in a magnitude estimation task (Eerland et al., 2011), and the current situation/context influence how we process magnitude/numbers. The lateral head turns in Loetscher et al.’s (2008) study and ours and the arm turns in our study are all situation-/context- based tasks. Similar to those tasks, in daily life we frequently rotate our head or turn arms in response to a specific situation of the environment and/or particular contextual requirement of a task. Our results provided the first evidence that two sensorimotor-based situated factors, such as a lateral head turn and a lateral arm turn, can cancel each other out regarding their respective influence on numerical cognition.

The situatedness of SNAs in head and arm turns revealed by the present study is also in line with many recent findings demonstrating flexible, context/situation-dependent SNARC effects. For example, by asking participants to mentally arrange numbers along a ruler (on which smaller numbers are located on the left and larger numbers on the right), a typical SNARC pattern was observed (Bächtold et al., 1998). However, a reversed SNARC pattern was found when the participants were required to mentally arrange numbers along a clock face (on which smaller numbers are located on the right and larger numbers on the left). The flexibility of the SNARC directions was also revealed by later studies that manipulated most recent reading experience. For example, Shaki and Fischer (2008) found that a group of bilingual Russian-Hebrew speakers showed a significant SNARC effect after reading a Russian text (left-to-right reading direction) for 10 min, whereas the SNARC magnitude significantly reduced after reading a Hebrew text (right-to-left reading direction) for the same amount of time. Similarly, Fischer et al. (2010) systematically manipulated horizontal positions of a small number and a large number inside novel cooking recipes in both English and Hebrew. They found that the magnitudes and directions of SNARC effects were influenced by reading directions (English vs. Hebrew) as well as the positions of the two numbers, i.e., congruent (smaller numbers on the left and larger numbers on the right) or incongruent (smaller numbers on the right and larger numbers on the left) with the SNARC effect. Compared with these studies manipulating either visual imagery instructions or recent reading experience on SNARC effects, the present study manipulating sensorimotor body experience on RNG tasks revealed different aspects for the situatedness of SNAs. The SNARC-based studies may imply that the orientation of the MNL in a particular task is situation/context- dependent, i.e., the origin of the MNL is likely related with a working memory-based mechanism (van Dijck and Fias, 2011) as well as a long term memory-based mechanism (Dehaene et al., 1993). Whereas our study emphasized that the accessibility of the numerical representation along the presumed MNL is situation/context- dependent and can be altered in a trial by trial basis.

The most recent progress to the hierarchical model is the Tropic, Embodied, and Situated Theory (TEST) of cognition (Myachykov et al., 2014). This model introduces the distinctions of online (e.g., simulated) processing vs. oﬄine (stored) representations into the hierarchical model and further emphasizes how these distinctions lead to a dynamic relationship between the tropic (reflecting features and constraints of the physical world; replacing the term ‘grounded’ as proposed by Myachykov et al., 2014), embodied, and situated representational features. A natural prediction following the TEST (Myachykov et al., 2014) is that, the hierarchal relationship between embodied and situated processing may depend on the dynamic balance between stored representations and online processing. The effect of the relative depth of processing can certainly shed some light on how to explain the situatedness of SNAs found in the abovementioned SNARC-based studies and in the present study. In those studies, visual imagery instructions (Bächtold et al., 1998), manipulation of recent reading experience (Shaki and Fischer, 2008; Fischer et al., 2010), or body movements (Loetscher et al., 2008) may all elicit deeper online (e.g., simulated) processing rather than the oﬄine (stored) knowledge representations. The deeper online processing in either a SNARC or RNG paradigm may lead to a relatively higher activity of the situated spatial representations of numbers, compared with those already stored (oﬄine) spatial representations of numerosity.

The situatedness of SNAs was also observed beyond the SNARC effect. For example, a recent study (Wasner et al., 2014) demonstrated that the finger counting preference is consistently influenced by situated factors. These factors include influence from perception, i.e., visual inputs through the horizontal (left-to-right) perceptual arrangement of displayed fingers and instruction, and influence from proprioception, i.e., sense for postures and locations elicited by one hand busy in writing prior to counting (Wasner et al., 2014). Interestingly, quantitative estimations to the Eiffel Tower’s height are smaller when healthy participants’ bodies are leaning to the left than to the right (Eerland et al., 2011). Similarly, a most recent study (Göbel, 2015) demonstrated that the most recent reading experience influenced subsequent counting behavior. By carrying out two experiments, the present study revealed that the numerical representations can be dynamically modulated by two sensorimotor-based situated factors and that these two situated factors can interact with each other. Combining previous findings based on the SNARC effect, finger counting, and body postures, and our findings based on body movements, all the existing empirical data provided strong support to the notion of fragile and context-dependent SNAs and suggested that the SNAs can be modulated by recent experience of spatial cues contained in either single or two situated factors.

### Sensorimotor Experience and Numerical Cognition

The present study added new evidence to the view that body motion can modulate numerical processing. Our study integrated very well with a growing body of literature demonstrating numerical effects in different sensorimotor circumstances or different effectors, including body posture (Eerland et al., 2011), random walk (Shaki and Fischer, 2014), skin pressure (Holmes and Lourenco, 2013), lateral head turns (Loetscher et al., 2008; Winter and Matlock, 2013), lateral eye turns (Loetscher et al., 2010), optokinetic stimulation (OKS)-induced eye movements (Ranzini et al., 2014), cross-modal tactile information (Krause et al., 2013), and finger counting (Fischer and Brugger, 2011).

Particularly, our findings provided new piece of evidence for the close links between arm/hand-based sensorimotor activities and numerical processing (Butterworth, 1999; Simon, 1999; Fischer, 2003; Andres et al., 2004; Badets et al., 2007, 2010; Brozzoli et al., 2008; Badets and Pesenti, 2010; Klein et al., 2011; Tschentscher et al., 2012; Wasner et al., 2014). For example, it has been suggested that numbers are associated with fingers since brain regions involved during numerical processing of approximate quantities (i.e., the intraparietal sulci of the left and right parietal lobes) are also part of the neutral circuit controlling handshapes and finger movements during finger counting (Butterworth, 1999; Simon, 1999). An automatical association of numbers and fingers was also revealed in both behavioral (Klein et al., 2011) and neural (Andres et al., 2007; Sato et al., 2007; Tschentscher et al., 2012) studies. Some researchers (Fischer and Brugger, 2011) even suggested that the origin of the SNAs is likely due to hand-based sensorimotor experience, such as finger counting. Another research line on grasping also indicated a close relationship between sensorimotor activities and numerical processing in effectors of arms and hands, which is more closely related with our findings of the present study. With accumulating evidence, a bidirectional association was revealed. The representation of number magnitude can influence grasping actions (Andres et al., 2004; Badets et al., 2007) and the modulation in an opposite direction is also true (Badets and Pesenti, 2010; Badets et al., 2010, 2012). The third evidence came from the effect of observed human pointing movements. A recent study (Badets et al., 2015) demonstrated that mere observations of perceptual cues of mimicking human pointing movements of the forefinger can affect performance on a subsequent RNG task. This result is in line with earlier studies in that space-number biases can be induced by various perceptual cues, including observations of the leftward or downward oriented gazes on a screen (Grade et al., 2013) and observations of a closing or opening grip of human hands (Badets et al., 2012).

The abovementioned studies are largely based on perceptual and/or static cues of hands, local movements of fingers in a small scale, or observations of arm or hand/finger movements. Instead, our findings in the present study demonstrated a close linkage between the numerical magnitude and arm movements of a large magnitude relative to the body trunk and revealed an arm-based sensorimotor bias to internal numerical representations in a RNG task. Taken together, the present study extended the current literature, especially regarding associations between arm/hand-based sensorimotor activities and numerical processing.

### Proprioceptive, Vestibular Activities, and Numerical Cognition

In many situations, our brain must rely on multiple sensorimotor activities, including those from the vestibular system and those from the proprioceptive system, to estimate the position, movement, and acceleration of the body from moment to moment. Thus, it is intriguing to unravel the exact nature of the influence from both the vestibular and proprioceptive systems on high level processing, such as numerical cognition. By using a paradigm involving motion platform, Hartmann et al. (2012) revealed that the bottom–up vestibular activation alone, rather than additional action planning or motor activity, can induce numerical biases (but see Shaki and Fischer, 2014). However, it is still unknown whether the involvement of the vestibular system is a prerequisite for inducing the instantaneous numerical biases in situations when relative movements between participants and the external world exist and the accompanying sensorimotor activity, rather than mere perceptual cues, are involved. Particularly, in these situations, can inputs from the proprioceptive system *per se*, without estimation of the motion (linear and angular accelerations) of the head in space based on processing of vestibular sensorimotor activities, produce a similar numerical bias as inputs from the vestibular system?

As briefly mentioned in the introduction, we argued that the vestibular system is not explicitly influenced by lateral turns of both arms^3^, given the obvious activation of the proprioception system, i.e., estimation of the perceived positions of one’s arms relative to one’s own body based on processing of proprioceptive sensorimotor activities (Smeets et al., 2006). In contrast, lateral head turns activate the proprioceptive system as well as the vestibular system by stimulating neck muscle proprioception, the semicircular canal system and the otoliths together (Day and Fitzpatrick, 2005). According to the results of Experiment 1, sensorimotor activities from the proprioceptive system alone, e.g., those generated by lateral arm movements, can bias the instantaneous random number production and exert influence similar to that on lateral head turns when the vestibular and proprioceptive systems are activated simultaneously. Thus, our study may shed some light on disentangling proprioceptive and vestibular contributions to SNAs and imply that proprioceptive inputs *per se*, rather than additional vestibular activation, are able to produce numerical biases.

We acknowledge the validity of the attentional account in explaining the findings of the current literature and of our study, i.e., a common attentional mechanism is underlying numerical biases induced by both lateral head turns in Loetscher et al.’s (2008) study and lateral arm turns in the present study. In fact, our paradigm itself cannot disentangle the attentional mechanism (Loetscher et al., 2008) from the proprioceptive contribution. Either of them may lead to biases in numerical cognition. It is not yet clear and still possible that moving one’s arm to the left in our task (even when eyes were covered by a mask) might lead to certain degree of shift of spatial attention to the left side, similar to that in Loetscher et al.’s (2008) task. Meanwhile, some other factors, such as motor planning or intention to move (Shaki and Fischer, 2014), might be able to induce potential shift of spatial attention in situations of either head or arm movements (but see Hartmann et al., 2012). Thus, more research is needed to determine the exact nature of the proprioceptive contribution and the potential causality between attention shifts and proprioceptive activations.

Our explanation based on the proprioceptive contribution to RNG biases is not necessarily incompatible with Hartmann et al.’s (2012) finding that vestibular activation itself can elicit numerical biases. Hartmann et al.’s (2012) study and ours might reveal different aspects of how sensorimotor activities are dynamically involved in our instantaneous numerical cognition. Finally, based on our findings, it might be interesting to ask further whether more complex activities consisting of composite-body-movements, such as yoga practice and martial arts, might exert similar influence in shaping the mind’s momentary abstract thought.

## Ethical Approval

All procedures performed in studies involving human participants were in accordance with the ethical standards of the institutional and/or national research committee, the American Psychological Association (APA) standards and with the 1964 Helsinki declaration and its later amendments or comparable ethical standards.

## Conflict of Interest Statement

The authors declare that the research was conducted in the absence of any commercial or financial relationships that could be construed as a potential conflict of interest.

## Footnotes

1 We thank the second reviewer for providing this idea that natural orienting responses and compensatory postural reflexes offer instances of congruent and incongruent head and body movements, respectively.

2 These conditions were named with reference to the head turn direction.

3 In fact, effects in the opposite direction were observed in the recent literature, i.e., the vestibular signals induced by externally-driven (i.e., passive) whole-body displacements contribute to the control of arm movement (Raptis et al., 2007) and the vestibular signals induced by galvanic vestibular stimulation (GVS) modulate the proprioception of the forearms (Schmidt et al., 2013).
